# Fetal congenital heart disease - mode of delivery and obstetrical complications

**DOI:** 10.1186/s12884-022-04910-w

**Published:** 2022-07-19

**Authors:** Keren Zloto, Alyssa Hochberg, Kinneret Tenenbaum-Gavish, Alexandra Berezowsky, Shiri Barbash-Hazan, Ron Bardin, Eran Hadar, Anat Shmueli

**Affiliations:** 1 Sackler Faculty of Medicine, Tel Aviv University, Tel Aviv, Israel; 2 Helen Schneider Hospital for Women, Rabin Medical Center, 39 Jabotinsky St., 4941492 Petach Tikva, Israel

**Keywords:** Congenital heart diseases, Operative vaginal delivery, Non-reassuring fetal heart rate

## Abstract

**Background:**

The optimal mode of delivery in cases of fetal congenital heart disease (CHD) is not established. The few relevant studies did not address operative vaginal delivery. The aim of this study was to assess the impact of fetal CHD on mode of delivery during a trial of labor, and to secondarily describe some obstetric complications.

**Methods:**

The database of a tertiary medical center was searched for women who gave birth to a singleton, liveborn neonate in 2015–2018. Mode of delivery was compared between women carrying a fetus with known CHD and women with a healthy fetus matched 1:5 for maternal age, parity, body mass index, and gestational age.

**Results:**

The cohort included 616 women, 105 in the CHD group and 511 in the control group. The rate of operative vaginal delivery was significantly higher in the CHD group (18.09% vs 9.78%, OR 2.03, 95% CI 1.13–3.63, *p* = 0.01); the difference remained significant after adjustment for nulliparity and gestational age at delivery (aOR 2.58, 95% CI 1.36–4.9, *p* < 0.01). There was no difference between the CHD and control group in rate of intrapartum cesarean delivery (9.52% vs 10.76%, respectively, OR 0.97, 95% CI 0.47–1.98, *p* = 0.93). The most common indication for operative vaginal delivery was non-reassuring fetal heart rate (78.94% vs 64%, respectively). Median birth weight percentile was significantly lower in the CHD group (45th vs 53rd percentile, *p* = 0.04).

**Conclusions:**

Our findings suggest that operative vaginal delivery, performed mostly because of non-reassuring fetal heart rate, is more common in pregnancies complicated by a prenatal diagnosis of CHD than non-anomalous pregnancies.

## Background

Congenital heart diseases (CHDs) are the most common fetal structural anomalies, affecting nearly 1% of births annually in the United States [1–4]. During the last decades, antenatal diagnosis of cardiac anomalies has increased due to enhanced prenatal imaging including fetal sonography and echocardiography [5–7].

Most fetal CHD studies are grouped by the time of diagnosis, namely, before or after delivery [2, 7–12]. With prenatal diagnosis, delivery can be planned for a tertiary center where the expected cardiac intervention will be performed following prompt multidisciplinary evaluation by the appropriate cardiac, surgical, and neonatal staff [8, 11]. However, the optimal mode of delivery in cases of fetal CHD is not well determined [10]. The presumed neonatal advantage of cesarean delivery (CD) has yet to be established and weighed against the potential maternal risk [8]. Comparisons of women with a prenatal or postnatal diagnosis of CHD showed that prenatal diagnosis decreased the likelihood of spontaneous labor [10] and increased the likelihood of a planned CD [2, 10, 13]. Nevertheless, a practice of elective CD was not found to be associated with decreased neonatal morbidity and mortality compared to attempted vaginal delivery; rather, it was associated with both higher rates of maternal morbidity as well as longer maternal and neonatal hospitalization [13–16]. In contrast, several studies demonstrated that a prenatal diagnosis of CHD increased the odds of induction of labor but not of CD [12, 17]. Levi et al. [17] found no significant difference in CD rate between women with a prenatal or postnatal diagnosis of fetal CHD.

Few studies have compared obstetrical and outcome parameters of pregnancies complicated by fetal CHD with normal pregnancies. Some suggested that mothers carrying fetuses with CHD were at increased risk for CD [18–20] and instrumental delivery [20]. Ge et al. [19] found that the most common indication for CD in this patient group was non-reassuring fetal heart rate (NRFHR).

Most studies of the mode of delivery in women diagnosed with fetal CHD compared CD to vaginal delivery, without considering operative vaginal delivery (OVD) and without detailing the indications for such interventions. The aim of the present study was to determine whether fetal CHD affects the mode of delivery during a trial of vaginal delivery.

## Methods

### Study population

For the present retrospective cohort study, the healthcare database of a tertiary medical center was searched for all women with a singleton gestation who gave birth between March 2015 and July 2018. Women carrying a fetus with known CHD were identified and formed the study group. The control group was randomly selected from among the women who gave birth to a healthy liveborn neonate and were matched 1:5 with the study group for maternal age, body mass index, parity, and gestational age at delivery. Fetal CHDs were identified using the coding criteria of the International Classification of Diseases, Ninth Revision (ICD-9) and categorized as minor or major according to the Centers for Disease Control and Prevention (CDC) classification [21]. We further divided them into cyanotic or non-cyanotic according to the commonly accepted classification in the literature [22].

Exclusion criteria were elective CD, termination of pregnancy, intrauterine fetal death, and other non-cardiac congenital anomalies, either chromosomal or genetic abnormalities or anatomical malformations. We also excluded cases of minor cardiac anomalies such as patent ductus arteriosus and patent foramen ovale as well as congenital valve disease and hypoplastic heart syndrome. In our center, fetal monitoring is not performed in women carrying a fetus with suspected hypoplastic heart syndrome, because the mode of delivery does not affect prognosis [23]. Cases of multiple fetal cardiac defects were included in the major cardiac anomaly category.

### Data collection

Data for the study were retrieved from the comprehensive computerized perinatal database of our center. The collected data included demographics (maternal age, body mass index, smoking status), clinical-obstetrical parameters (gravidity, parity, prior CD, chronic disease and medication use), chronic diseases (antiphospholipid antibody syndrome, asthma, epilepsy, inherited thrombophilia, type 1 and type 2 diabetes mellitus, hypothyroidism, hyperthyroidism, and chronic hypertension), pregnancy complications [gestational diabetes, gestational hypertensive disorders in pregnancy - pre-eclampsia, hemolysis, elevated liver enzymes and low platelet count syndrome (HELLP), cholestasis of pregnancy, polyhydramnios, oligohydramnios, chorioamnionitis, placental abruption, NRFHR, postpartum hemorrhage, shoulder dystocia, adherent placenta, and placenta previa], onset of labor (spontaneous or induced), mode of delivery, and indication for intervention (CD or OVD). Short-term perinatal data included gestational age at birth, sex, birth weight, meconium-stained amniotic fluid, and APGAR score.

### Outcome measures

The primary outcome measure of the study was mode of delivery: spontaneous vaginal delivery, OVD, or CD. OVD and CD were further categorized by indication for intervention: NRFHR or prolonged second stage of labor, and maternal exhaustion for OVD; NRFHR or arrested/protracted labor for CD.

The secondary outcome measures were birth weight and birth weight percentile according to the Israeli national birthweight curves, [24] small for gestational age (defined as birth weight below the 10th percentile), large for gestational age (defined as birth weight above the 90th percentile), meconium-stained amniotic fluid, and APGAR score 7 or lower.

Induction of labor was achieved with either prostaglandin E2 vaginal inserts, extra-amniotic balloon, or amniotomy and oxytocin infusion. The specific method was left to the discretion of the physician.

Spontaneous vaginal delivery was defined as any vaginal delivery not assisted by vacuum extraction of forceps. OVD (Operative Vaginal Delivery) was defined as any vaginal delivery assisted by either vacuum delivery or forceps.

### Statistical analysis

Continuous variables were evaluated for normal distribution using histograms and Q-Q plots. Normally distributed continuous variables were described as mean and standard deviation (SD), and non-normally distributed continuous variables, as median and interquartile range (IQR). Categorical variables were described as frequency and percentage. Continuous variables were compared using Mann–Whitney test. Correlations between continuous variables were evaluated using the Spearman correlation coefficient. Chi-squared test was applied to compare categorical variables. Multivariate logistic regressions were used to evaluate the association between fetal CHD and MOD, chorioamnionitis, NRFHR, 1-minute and 5-minute APGAR scores ≤7, small for gestational age, and meconium-stained amniotic fluid. The multivariate models were performed twice. The model included nulliparity, mode of delivery, and gestational age at birth as confounders. Odds ratios (OR) and 95% confidence intervals (CI) were calculated. All statistical tests were two-tailed, and *p* < 0.05 was considered statistically significant. Data were generated with SPSS version 26.0 (IBM, Chicago, IL, USA).

## Results

Of the 237 women diagnosed with fetal CHD during the study period, 105 were eligible for the study (CHD group). The main reasons for exclusion were patent ductus arteriosus (*n* = 40), patent foramen ovale (*n* = 36), and hypoplastic heart syndrome (*n* = 20), followed by pulmonic stenosis (*n* = 16), tricuspid regurgitation (*n* = 7), aortic stenosis (*n* = 5), and mitral regurgitation (n = 1). All diagnoses were made prenatally. The most common major cardiac defect was coarctation of the aorta)25.71% of total CHDs). Other cardiac anomalies included transposition of the great arteries (22.85%), ventricular septal defect (21.9%), tetralogy of Fallot (15.23%), atrial septal defect (7.61%), Ebstein anomaly (4.76%), and atrioventricular septal defect (1.9%). The control group consisted of 511 women carrying a healthy singleton fetus within the same period.

The baseline demographic, obstetrical, and clinical characteristics of the cohort are summarized in Table 1. There were no between-group differences in maternal age, body mass index, parity, and gestational age at delivery, as dictated by the matching process. Rates of prior CD were similar in the two groups. A significantly higher proportion of women in the CHD group were smokers (4.76% vs 1.17%, *p* = 0.01). There was no significant difference between the groups in the rate of chronic diseases (20.95% vs 15.68%, *p* = 0.18), although a higher proportion of women in the CHD group had hypothyroidism (9.52% vs 3.52%, *p* < 0.01).

**Table 1:** Baseline demographic, clinical, and obstetrical characteristics of women carrying a fetus with CHD compared to women with a non-anomalous pregnancy

Characteristic	CHD ***n*** = 105	Non-CHD ***n*** = 511	***p***-value
Maternal age, years	30 (27–33.5)	30 (26–33)	0.2
Gravity	2 (1–4)	2 (1–3)	0.09
Parity	1 (0–2)	1 (0–2)	0.36
Nulliparity	38 (36.1%)	203 (39.7%)	0.5
Prior cesarean delivery	0 (0–0)	0 (0–0)	0.43
Body mass index, Kg/m^2^	22.18 (20.2–25.7)	22.23 (20.3–24.3)	0.58
Smoking	5 (4.7%)	6 (1.1%)	0.01
Chronic medication use	20 (19.%)	59 (11.5%)	0.03
Chronic disease:	22 (20.9%)	80 (15.6%)	0.18
APLA syndrome	0 (0%)	2 (0.3%)	0.52
Asthma	0 (0%)	9 (1.%)	0.17
Epilepsy	2 (1.9%)	5 (0.9%)	0.41
Inherited thrombophilia	2 (1.9%)	5 (0.9%)	0.41
T1DM/T2DM	11 (10.4%)	44 (8.6%)	0.07
Hypothyroidism	10 (9.5%)	18 (3.5%)	< 0.01
Hyperthyroidism	1 (0.9%)	4 (0.7%)	0.86
Chronic hypertension	5 (4.7%)	32 (6.2%)	0.55

Continuous variables are presented as median and interquartile range and categorical variables are presented as number and percent

*APLA* Antiphospholipid antibody [syndrome], *CHD* Congenital heart disease, *T1DM* Type 1 diabetes mellitus, *T2DM* Type 2 diabetes mellitus

The obstetrical outcomes are presented in Table 2. There were no between-group differences in rates of gestational diabetes, hypertensive disorders of pregnancy, amniotic fluid disorders, and postpartum hemorrhage. The CHD group had a significantly higher rate of NRFHR (21.9% vs 13.3%, OR 1.82, 95% CI 1.07–3.1, *p* = 0.02) and chorioamnionitis (3.8% vs 0.78%, OR 5.02, CI 1.23–20.4, *p* = 0.01). Both these differences remained significant after adjustment for gestational week at delivery (aOR 1.84, 95% CI 1.08–3.135, p = 0.02; aOR 5.87, 95% CI 1.41–24.41, p = 0.01).

**Table 2:** Maternal and obstetrical outcomes of pregnancies complicated by fetal CHD compared to non-anomalous pregnancies

Outcome	CHD ***n*** = 105	Non-CHD ***n*** = 511	***p***-value
Gestational diabetes	15 (14.2%)	49 (9.6%)	0.15
Gestational hypertension	2 (1.9%)	7 (1.3%)	0.67
Preeclampsia	5 (4.%)	11 (2.1%)	0.12
HELLP syndrome	0 (0%)	2 (0.4%)	0.52
Shoulder dystocia	1 (0.9%)	1 (0.2%)	0.21
Adherent placenta	3 (2.%)	4 (0.7%)	0.06
Placenta previa	0 (0%)	6 (1.1%)	0.26
Placental abruption	0 (0%)	6 (1.1%)	0.26
Chorioamnionitis	4 (3.8%)	4 (0.%)	0.01
Postpartum hemorrhage	8 (7.6%)	26 (5.%)	0.3
NRFHR	23 (21.9%)	68 (13.3%)	0.02
Oligohydramnios	4 (3.8%)	29 (5.6%)	0.43
Polyhydramnios	1 (0.9%)	12 (2.3%)	0.36
Cholestasis of pregnancy	0 (0%)	3 (0.5%)	0.43
Onset of labor			
Spontaneous	43 (40.9%)	235 (46%)	0.34
Induction	62 (59.%)	276 (54%)	
Mode of delivery			
Vaginal	76 (72.3%)	406 (79.4%)	0.04
Assisted vaginal	19 (18.1%)	50 (9.7%)	
Cesarean, intrapartum	10 (9.5%)	55 (10.7%)	

Continuous variables are presented as median and interquartile range categorical variables are presented as number and percent

CHD, congenital heart disease; HELLP, hemolysis, elevated liver enzymes, and low platelets; NRFHR, non-reassuring fetal heart rate

Regression analysis of the mode of delivery revealed a higher rate of OVD in the CHD group than the control group (18.09% vs 9.78%, OR 2.03, 95% CI 1.13–3.63, p = 0.01), although rates of CD were similar (9.52% vs 10.76%, OR 0.97, CI 0.47–1.98, *p* = 0.93). The association with OVD remained significant after adjustment for nulliparity and gestational age at delivery (OR 2.58, 95% CI 1.36–4.9, *p* < 0.01). The most common indication for OVD was NRFHR, with a higher prevalence in the CHD group (78.94% vs 64%), followed by prolonged second stage (21.05% vs 34%, respectively). Neither of these differences was statistically significant.

Perinatal outcomes are summarized in Table 3. There was no significant difference between the groups in newborn sex distribution (males: 56.19% vs 54.01%, *p* = 0.68). The CHD group was characterized by a significantly lower median birth weight percentile (45th vs 53rd percentile, *p* = 0.04) and, accordingly, a significantly higher frequency of small-for-gestational-age neonates (11.42% vs 5.87%, p = 0.04, OR 2.06, 95% CI 1.02–4.18). The association did not remain significant after adjustment for gestational age at delivery (aOR 1.97, 95% CI 0.96–4.04, *p* = 0.06). The CHD group also had higher rates of 1-minute and 5-minute APGAR scores ≤7 (15.42% vs. 2.94%, *p* < 0.01, OR 3.86, CI 1.72–8.66; 6.96% vs. 0.19%, *p* = 0.01, OR 7.47, 95% CI 1.23–45.27), but only the difference in 1-minute APGAR score < 7 remained significant after adjustment for mode of delivery and gestational week of delivery (aOR 4.12, 95% CI 1.74–9.78, *p* < 0.01; aOR 5.66, 95% CI 0.89–35.63, p = 0.06). The CHD group had a significantly higher rate of meconium-stained amniotic fluid on regression analysis (21.9% vs 11.74%, p < 0.01, OR 2.1, 95% CI 1.23–3.6) and after adjustment for gestational week of delivery (aOR 2.3, 95% CI 1.33–4, p < 0.01).

**Table 3:** Neonatal outcomes of pregnancies complicated by fetal CHD compared to non-anomalous pregnancies

Neonatal outcome	CHD ***n*** = 105	Non-CHD ***n*** = 511	***p***-value
Gestational age at delivery, week	39.1 (37.8–40.2)	39.2 (38.2–40.2)	0.49
Preterm birth < 34 gestational weeks	1 (0.9%)	4 (0.7%)	0.86
Preterm birth < 37 gestational weeks	13 (12.3%)	41 (8.%)	0.15
Neonatal gender, male	59 (56.2%)	276 (54.%)	0.68
Neonatal birthweight, grams	3126 (2837–3434)	3210 (2898–3504)	0.06
Neonatal birthweight, percentile	45 (27.5–69.5)	53 (31–75)	0.04
Meconium-stained amniotic fluid	23 (21.9%)	60 (11.7%)	< 0.01
Small for gestational age	12 (11.4%)	30 (5.8%)	0.04
Large for gestational age	5 (4.7%)	45 (8.8%)	0.16
One-minute APGAR ≤7	11 (15.4%)	15 (2.9%)	< 0.01
Five-minute APGAR ≤7	3 (6.9%)	2 (0.4%)	0.01

Continuous variables are presented as median and interquartile range categorical variables are presented as n (%)

CHD, congenital heart disease

Cyanotic heart defects (tetralogy of Fallot, transposition of the great arteries, Ebstein anomaly) were identified in 45 cases in the CHD group (Table 4). Comparison of this subgroup with the control group yielded no differences in baseline demographic, clinical, and obstetrical characteristics. On regression analysis, the cyanotic-CHD subgroup had a nearly significantly higher rate of OVD than the control group (20% vs 9.78%, OR 2.14, CI 0.97–4.74, *p* = 0.058) and a similar rate of CD (4.44% vs 10.76%, OR 0.43, CI 0.1–1.85, *p* = 0.26). After adjustment for nulliparity and gestational week of delivery, the association with OVD was significant (OR 2.6, 95% CI 1.06–6.35, *p* = 0.03).

**Table 4:** Maternal, obstetrical and neonatal outcomes of cyanotic CHD and non CHD

Outcome	Cyanotic CHD ***n*** = 45	Non-CHD (Controls) ***n*** = 511	***p***-value
GDM	5 (11.1%)	49 (9.6%)	0.74
Gestational HTN	1 (2.2%)	7 (1.3%)	0.64
Preeclampsia	1 (2.2%)	11 (2.1%)	0.97
HELLP Syndrome	0 (0%)	2 (0.4%)	0.67
Shoulder dystocia	0 (0%)	1 (0.2%)	0.76
Adherent	0 (0%)	4 (0.7%)	0.55
Previa	0 (0%)	6 (1.1%)	0.46
Abruption	0 (0%)	6 (1.1%)	0.46
Chorioamnionitis	0 (0%)	4 (0.7%)	0.55
Postpartum hemorrhage	4 (8.8%)	26 (5.%)	0.27
NRFHR	7 (15.5%)	68 (13.3%)	0.67
Oligohydramnios	3 (6.6%)	29 (5.6)	0.78
Polyhydramnios	0 (0%)	12 (2.3%)	0.3
Cholestasis of pregnancy	0 (0%)	3 (0.5%)	0.6
Onset of labor			
Spontaneous	15 (33.3%)	235 (45.9%)	0.10
Induction	30 (66.6%)	276 (54.%)	
Mode of delivery			
Vaginal (spontaneous)	34 (75.5%)	406 (79.4%)	0.056
Assisted vaginal (vacuum)	9 (20%)	50 (9.7%)	
Cesarean	2 (4.4%)	55 (10.7%)	
Pregnancy week	39.1 (37.5–40.5)	39.2(38.2–40.2)	0.93
Preterm birth33 > gestational weeks	0 (0%)	4 (0.7%)	0.55
Preterm birth < 37 gestational weeks	3 (6.6%)	41 (8%)	0.74
Neonatal gender, male	24 (53.3%)	276 (54%)	0.93
Neonatal birthweight, grams	3115(2855.5–3402)	3210 (2898–3504)	0.17
Neonatal birthweight, percentile	35 (24–68.5)	53 (31–75)	0.04
Meconium-stained amniotic fluid	9 (20%)	60 (11.7%)	0.1
SGA	5 (11.1%)	30 (5.8%)	0.16
LGA	3 (6.6%)	45 (8.8%)	0.62
APGAR 1 min < 7	5 (11.1%)	15 (2.9%)	0.005
APGAR 5 min < 7	0 (0%)	2 (0.39%)	0.67

Continuous variables are presented as median and interquartile range and categorical variables are presented as number and percent

*CHD* Congenital heart disease, *GDM* Gestational diabetes mellitus, *HELLP* Hemolysis, elevated liver enzymes, and low platelets, *HTN* Hypertension, *LGA* Large for gestational age, *NRFHR* Non-reassuring fetal heart rate, *SGA* Small for gestational age

## Discussion

Our results demonstrate that fetal CHD is associated with higher rate of OVD, indicated mainly for NRFHR, and has a negative impact on perinatal and obstetrical outcomes such as birth weight, APGAR score, and rates of meconium-stained amniotic fluid, chorioamnionitis, and NRFHR.

Previous studies have dealt with the importance of prenatal diagnosis of CHD [2, 7–12]. Whether the mode of delivery in these cases differs significantly from the general population remains unclear as do the indications for non-vaginal delivery. There is little mention in the literature of the rate of OVD and of intrapartum CD following a trial of labor. Hence our findings merit attention. Additionally, in contrast to the present study, the few studies that have addressed these issues [2, 7–12] compared the findings by time of diagnosis of CHD.

Our results are in line with the 2006 Swedish population-based study of 6343 fetuses with CHD [20]. The authors found that relative to the general population of pregnant women, women carrying a fetus with CHD had significantly higher rates of instrumental delivery (OR 1.21, 95% CI 1.10–1.34) and CD, either elective or non-elective (OR 1.91, 95% CI 1.79–2.03). However, the indications for instrumental delivery and CD were not evaluated.

Walsh et al., [18] using a similar study design to ours, compared women carrying a fetus with a major CHD to women with a non-anomalous singleton pregnancy and found that the study group had a higher rate of emergency CD, mostly attributable to NRFHR)10% vs 4.8%). The statistical significance was not maintained when the analysis was adjusted for the presence of major extracardiac anomalies. These findings were supported by another recent study demonstrating an increased risk for primary and non-primary CD in pregnancies complicated by CHD relative to non-anomalous pregnancies; again, the most common indication for primary CD was NRFHR (49% vs 24%) [19]. However, unlike the present study, these earlier investigations did not mention OVD. Furthermore, and more importantly, we included only fetuses with isolated CHD without major extracardiac malformations.

Indeed, we believe that our restricting the study group to cases of isolated CHD and excluding cases of elective CD may account for why the CD rate was not significantly higher in the CHD group than the control group (9.52 and 10.76%, respectively).

Our analysis of the obstetrical and perinatal outcomes of women with a diagnosis of fetal CHD is supported by earlier studies [18, 20, 25]. Walsh et al. [18] also showed that NRFHR is more common in cases of fetal CHD than in controls, and Cedergren et al. [20] reported a higher rate of meconium aspiration, fetal distress, and small-for gestational age among fetuses with CHD.

There are several potential mechanisms that might explain our results. First, fetuses with a cardiac anomaly are at increased risk of heart rate abnormalities and are therefore more prone to distress and, consequently, a higher likelihood of emergency delivery. Ueda al [24]. speculated that cardiac abnormalities are associated with abnormalities in fetal heart rate patterns and found that fetuses with CHD have a higher rate of emergency CD due to NRFHR. Second, fetuses with CHD may have lower tolerance and less reserve for labor, either owing to the cardiac anomaly itself or to overall smallness; therefore, their risk of OVD or CD due to NRFHR is higher [18]. Finally, as in the case of elective CD, physicians may tend to recommend emergency delivery due to their perception of fetal vulnerability. This is supported by several studies showing that a prenatal diagnosis of CHD could affect the mode of delivery and is associated with a higher rate of elective CD [2, 10].

The strength of the present study is the comparison of all modes of delivery, including OVD, between cases of isolated, severe fetal CHD and a control group without abnormalities. The mode of delivery in fetuses with CHD has hardly been investigated relative to the general population, and among the few studies of this issue, most did not consider OVD or the indications for CD or OVD**.** The present study is limited by the retrospective design with its inherent biases and the tertiary medical center setting which restricts the generalizability of the findings to all institutions. Another possible limitation of our study, possibly additionally restricting the generalizability of the findings, is our local practice to offer mothers of HLHS fetuses to avoid fetal monitoring during labor, which is not a common practice in other medical centers.

## Conclusions

Our study demonstrated that OVD, especially for an indication of NRFHR, is more common in pregnancies complicated by fetal CHD than in the general population, after exclusion of elective cesarean sections. In addition, fetal CHD was associated with higher rates of small for gestational age, meconium-stained amniotic fluid, 1-minute and 5-minute APGAR scores ≤7, NRFHR, and chorioamnionitis.

Physicians should be alerted to these findings and take them into consideration when deciding on mode of delivery. They may also be of help when counseling mothers with a prenatal diagnosis of CHD to regarding the possibility of a safe trial of labor. However, further large prospective studies are warranted.

## Data Availability

The datasets generated and/or analyzed during the current study are not publicly available, and will be available from the corresponding author on reasonable request after the approval of the local IRB, according to the Israeli and local IRB regulatory rules.
